# Supplementary material for: Konjac Ceramide (kCer)-Mediated Signal Transduction of the Sema3A Pathway Promotes HaCaT Keratinocyte Differentiation
Source: Biology (Basel). 2022 Jan 12;11(1):121. doi: 10.3390/biology11010121 (PMC8772740; doi:10.3390/biology11010121)
Supplement: Supplementary file 1 [file biology-11-00121-s001.zip › biology-1527303-supplementary.pdf]

biology

MDPI

Supplementary Figures

Konjac Ceramide (kCer)-mediated signal transduction of the Sema3A pathway promotes HaCaT keratinocyte differentiation

Seigo Usuki[1], Noriko Tamura[2], Tomohiro Tamura[2], Kohei Yuyama[1], Daisuke Mikami[1], Katsuyuki Mukai[1,3], Yasuyuki Igarashi[1]

[1]Lipid Biofunction Section, Frontier Research Center for Advanced Material and Life Science, Faculty of Advanced Life Science, Hokkaido University, Sapporo, Hokkaido, Japan

[2]National Institute of Advanced Industrial Science and Technology (AIST), Sapporo, Hokkaido, Japan.

[3]R&D Headquarters, Daicel Corporation, Tokyo, Japan

Corresponding author: Dr. Seigo Usuki, Lipid Biofunction Section, Frontier Research Center for Advanced Material and Life Science, Faculty of Advanced Life Science, Hokkaido University, Sapporo, Hokkaido, Japan Kita21, Nishi11, Kita Ward, Sapporo, Hokkaido, Japan 001-0021; tel: +81-11-706-9086; fax: +81-11-706-9024; e-mail: susuki@sci.hokudai.ac.jp

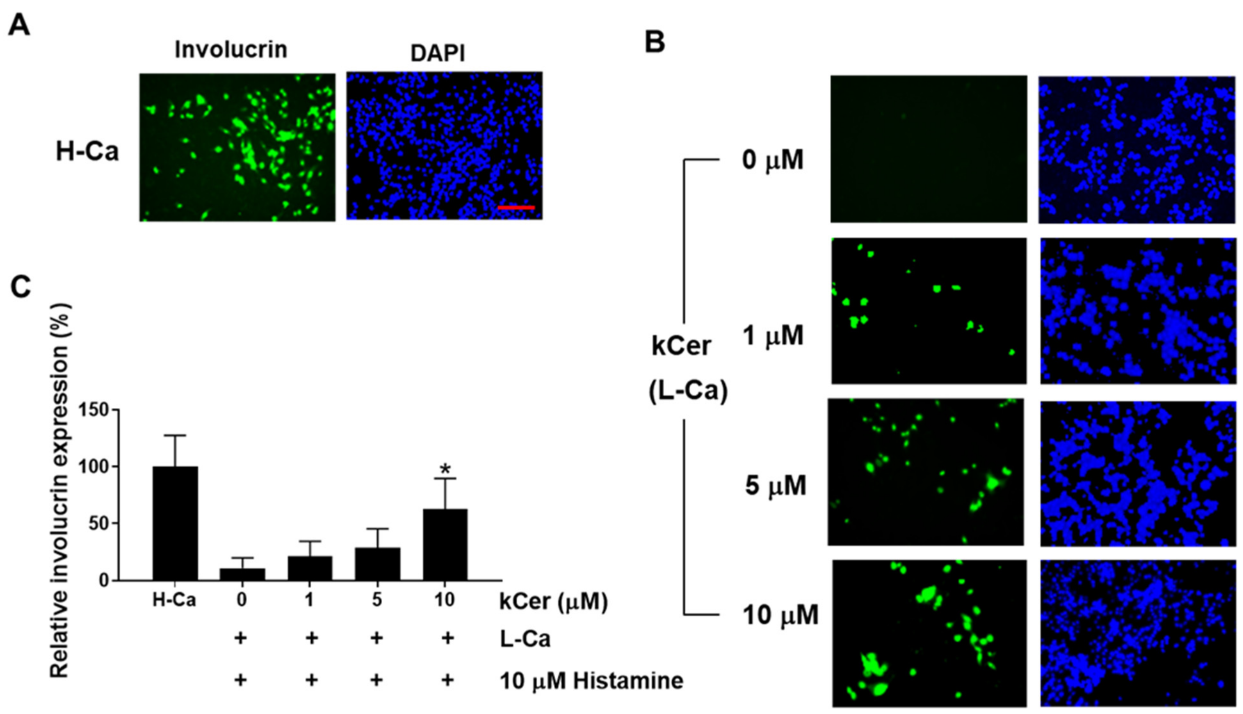

Figure S1.

kCer-induced HaCaT cell differentiation. Long-term cell culture was used for the HaCaT cell differentiation assay. After long-term L-Ca culture, cells were treated with (**A**) H-Ca medium or (**B**) L-Ca medium containing 0–10 μM kCer and 10 μM histamine. Left images in **A** and **B** are representative immunofluorescence images of involucrin staining. Right images show DAPI counterstaining. (**C**) Effect of kCer on HaCaT cell differentiation is shown as a graphic representation of relative involucrin expression, as measured by image J analysis of involucrin images shown in **A** and **B**. Data are presented as mean ± SD (n=3, *$p < 0.01$) by one-way ANOVA followed by Dunnett's test. Scale bar = 100 μm.

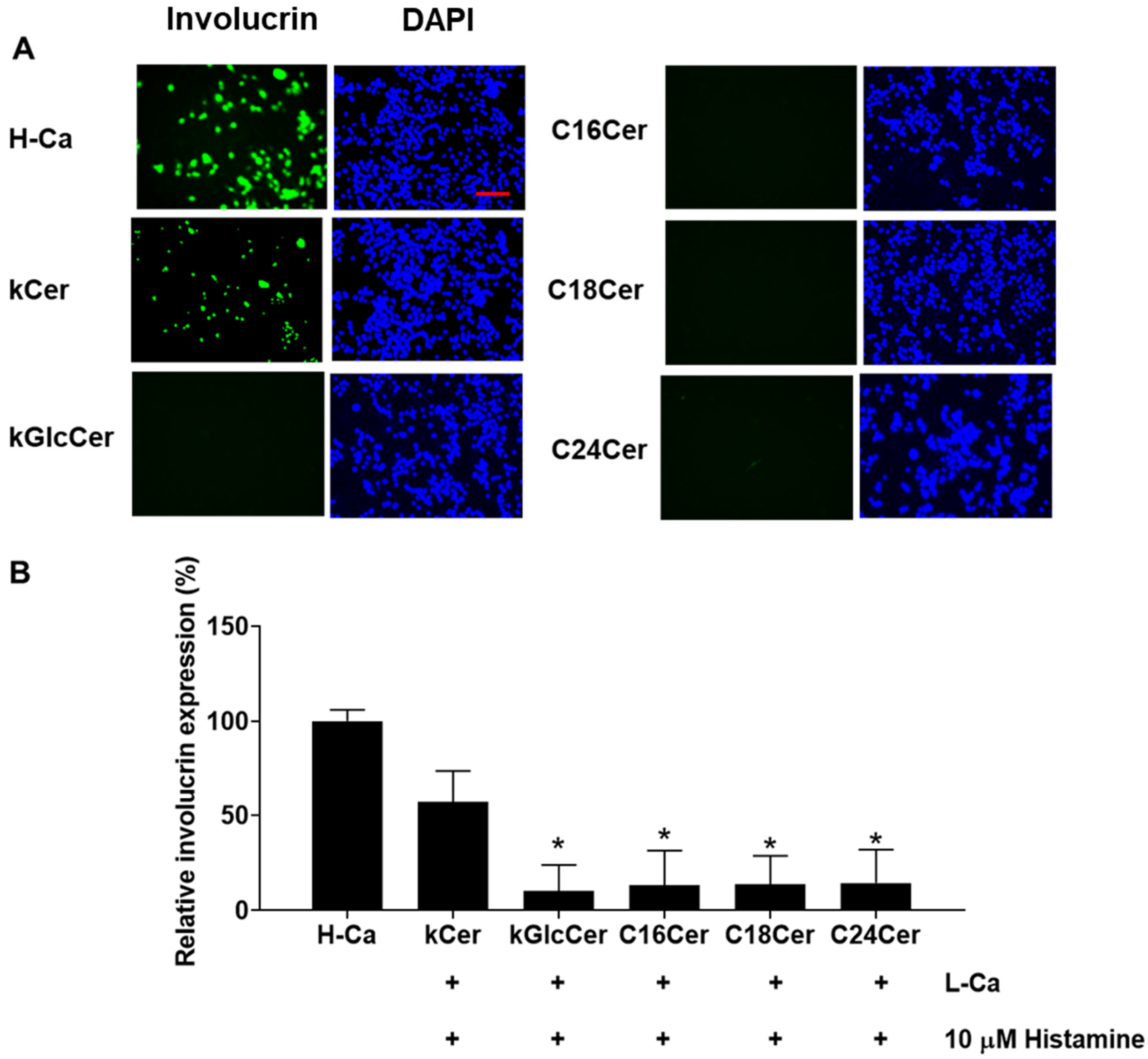

Figure S2.

Specificity of kCer-induced HaCaT cell differentiation activity. (**A**) Effects of kCer and other lipids (kGlcCer, C16Cer, C18Cer, and C24Cer) on HaCaT cell differentiation were compared by measuring relative involucrin expression using immunofluorescence staining with anti-involucrin antibody. Left images in **A** show representative involucrin immunofluorescence staining images, and right images show DAPI counterstaining. (**B**) Bar graph representation of the effects of kCer and other lipids on involucrin expression based on the results of immunofluorescence analysis represented in **A** is graphically presented. Data are presented as mean ± SD (n=3, *p<0.01) by one-way ANOVA followed by Dunnett's test. Scale bar = 100 μm.

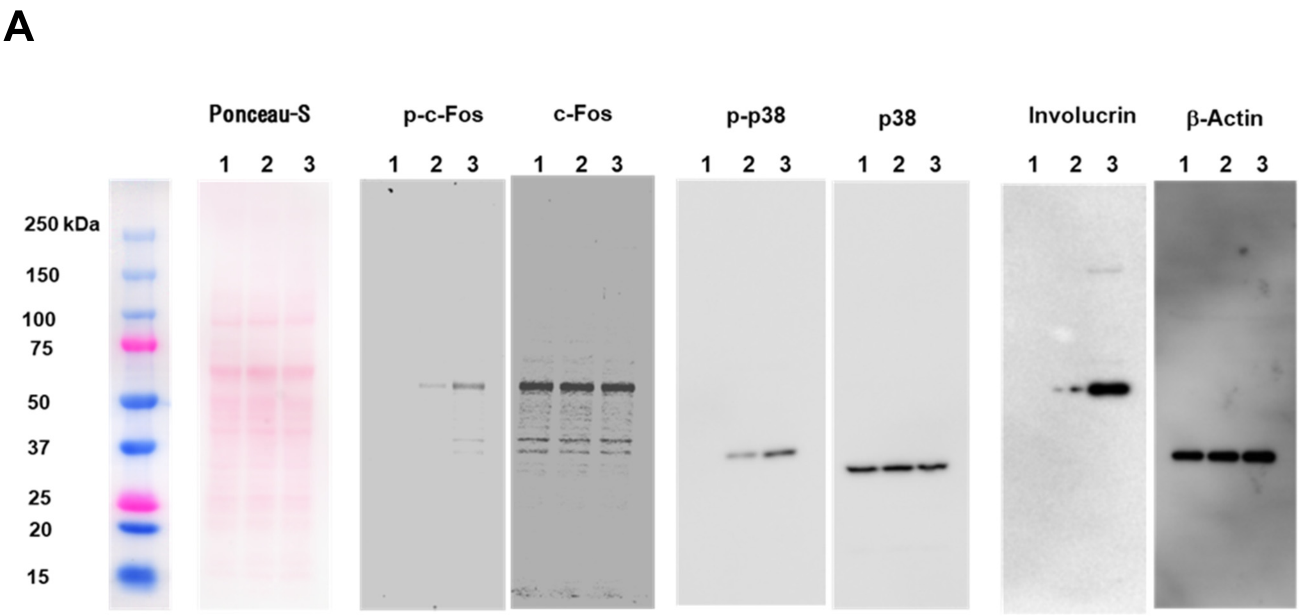

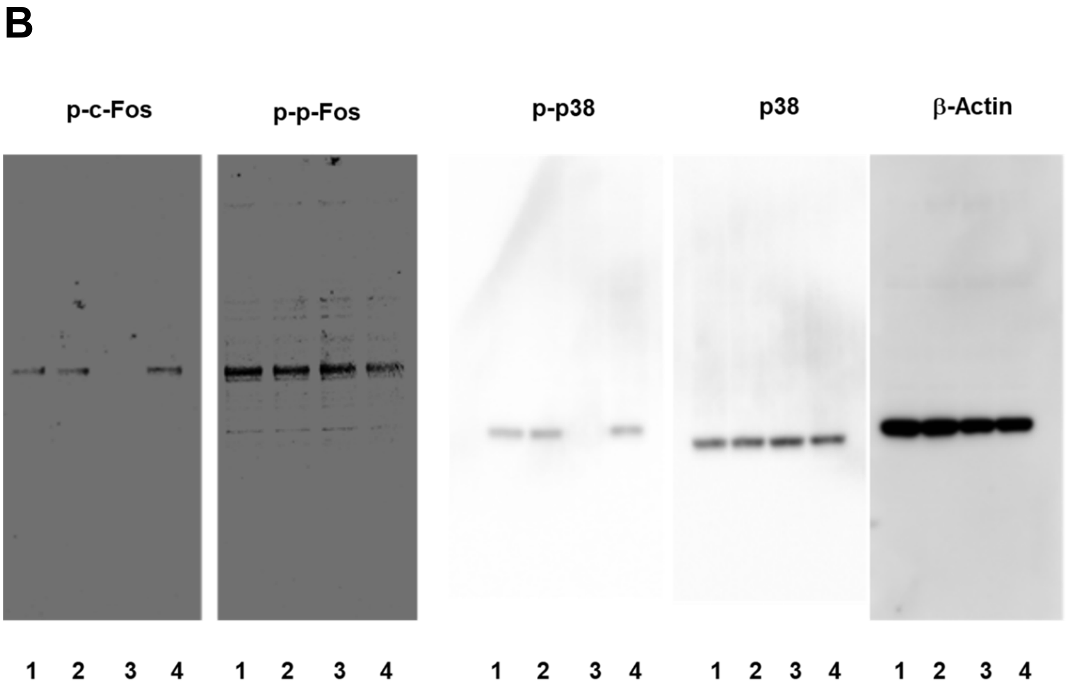

Figure S3.

Western blot membrane images used to generate Figure 3. **(A)** Lane 1, 2, and 3 correspond to 0, 1, and 10 µM kCer treatment, respectively. Protein levels of each of treatment are shown by Ponceau-S staining. **(B)** Lane1, 2, 3, and 4 corresponded to 10 µM kCer, 10 µM kCer+1 µM SB203580, 10 µM kCer+1 µM BIRB795, and10 µM kCer+1µM VX745, respectively.

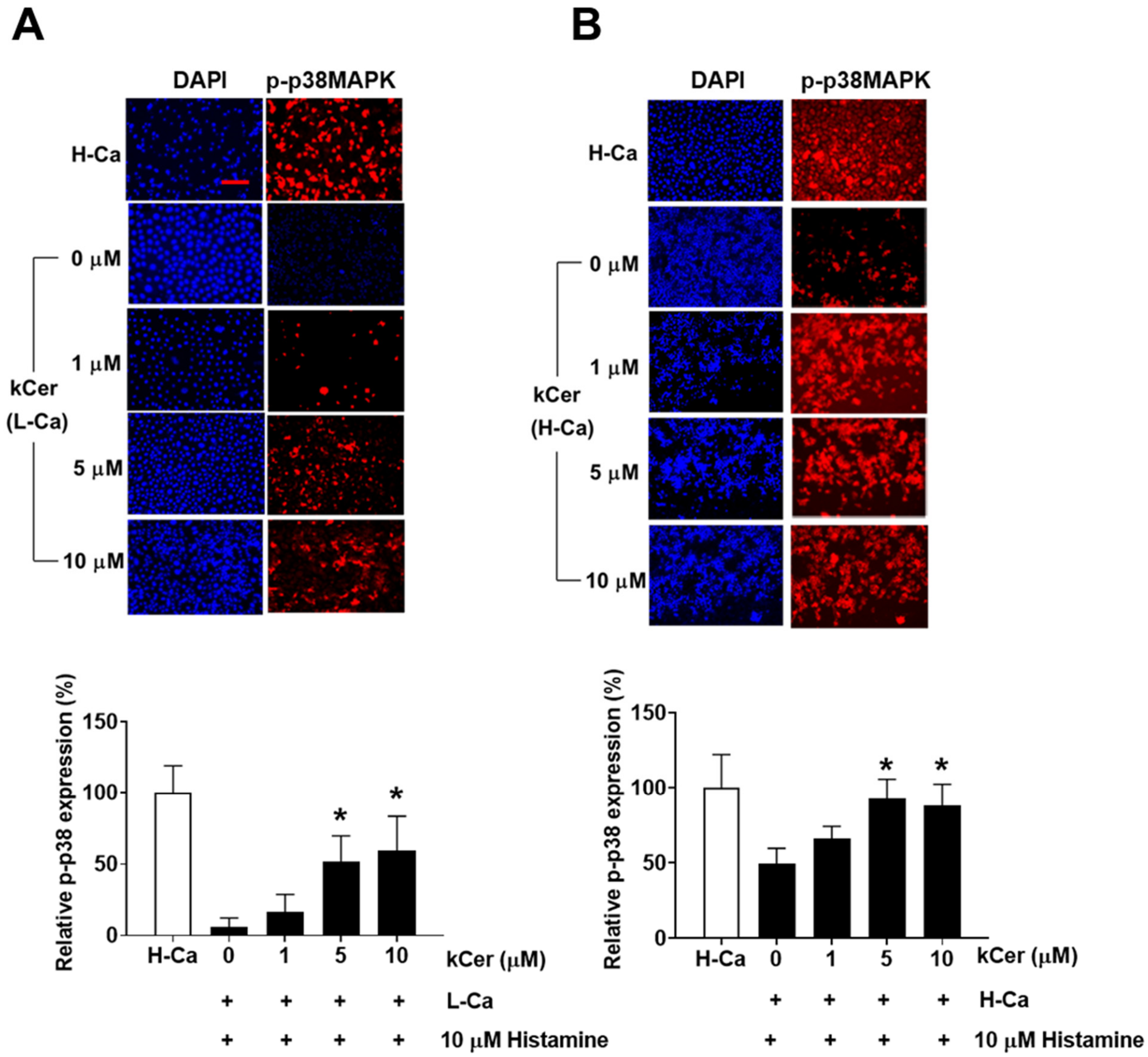

Figure S4.

kCer-induced p38MAPK activation in HaCaT cells. Effect of kCer on p38MAPK phosphorylation was examined after long-term culture with (**A**) H-Ca medium, or (**B**) L-Ca medium containing 0–10 μM kCer and 10 μM histamine. Right images in **A** and **B** are representative images of p-p38MAPK by immunofluorescence staining using a p-p38MAPK cell-based translocation assay kit. Left images in **A** and **B** show DAPI counter staining. Bar graphs of the effect of kCer on relative p-p38 expression are shown below the fluoresence images. The data for the bar graphs were obtained from the results of image J analysis shown in **A** and **B**. Data are presented as mean ± SD (n=3, *p<0.01). Scale bar = 100 μm.

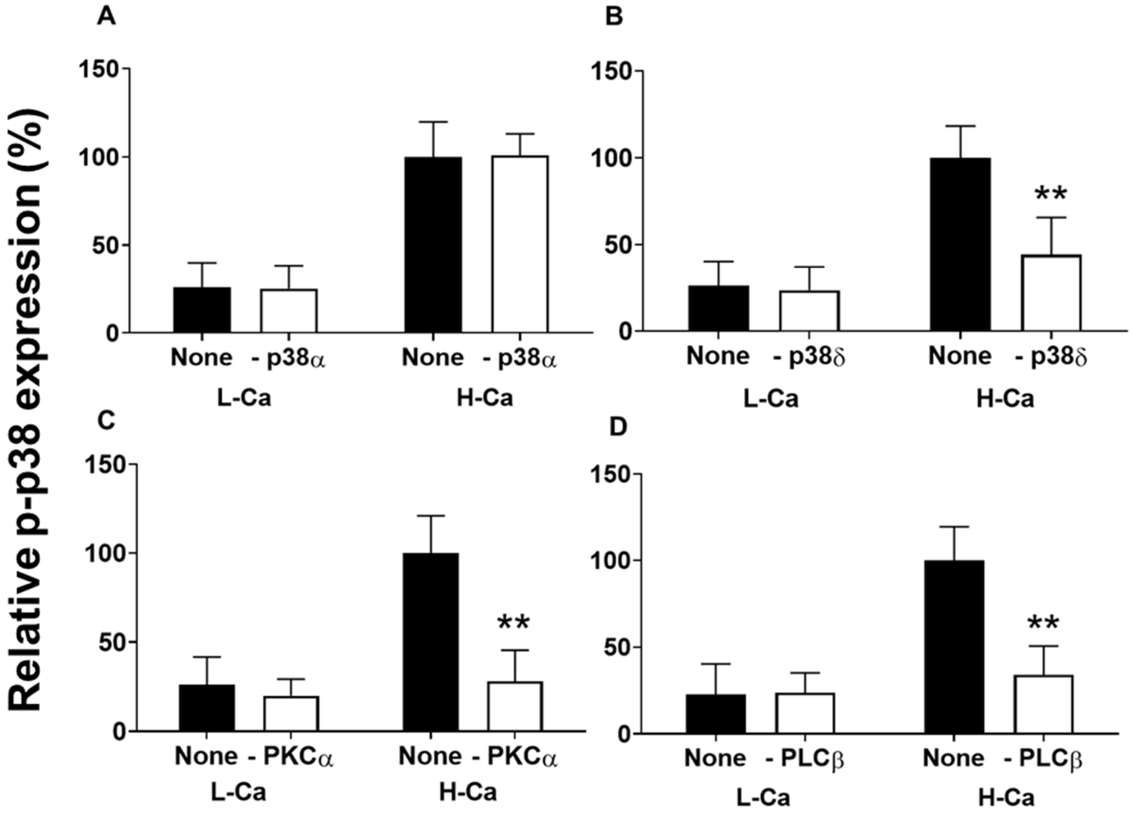

Figure S5.

Effects of siRNA on p-p38MAPK expression in differentiating cells treated with L-Ca or H-Ca medium. In L-Ca or H-Ca medium, undifferentiated cells were treated for 1 h with siRNA targeting p38 MAPK isoforms p38α or p38δ, PKCα, or phospholipase C (PLCβ), respectively, in **A**, **B**, **C**, and **D**. The cells were examined using a p-p38MAPK cell-based translocation assay kit. The results are presented as the means ± SD (n=3). **p<0.001 vs. no treatment (None), respectively, unpaired t-test.

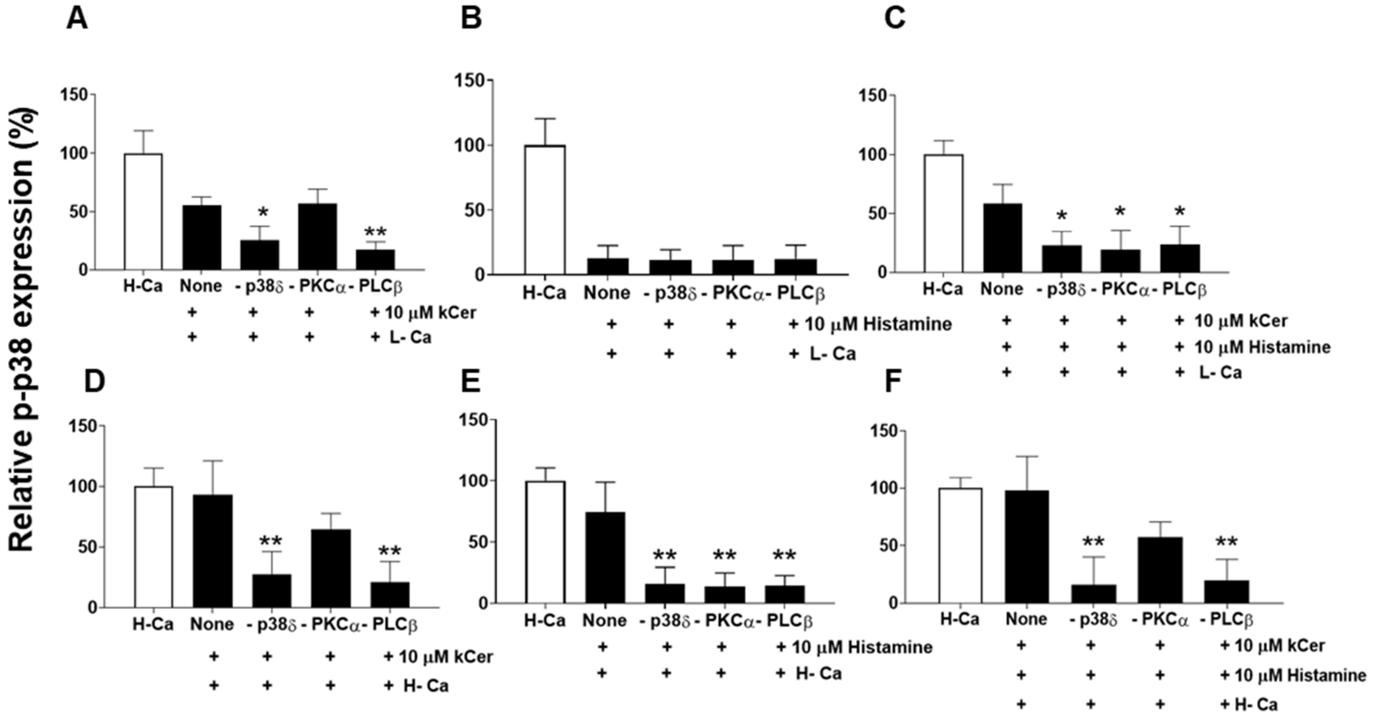

Figure S6.

Effects of siRNA on p-p38MAPK levels in cells differentiated or dedifferentiated by treatment with L-Ca (**A**, **B**, **C**) or H-Ca (**D**, **E**, **F**) medium. In L-Ca or H-Ca medium, undifferentiated cells were treated for 1 h with siRNA targeting p38δ, PKCα, or PLCβ in combination with 10 μM kCer (**A** and **D**), 10 μM histamine (**B** and **E**), or both in the presence of 10 μM kCer and 10 μM histamine (**C** and **F**). The cells were examined using a p-p38MAPK cell-based translocation assay kit. The results are presented as means ± SD (n=3). *p<0.01, **p<0.001 vs. no treatment (None), one-way ANOVA, followed by Tukey's multiple comparison post-test and Dunnett`s test.

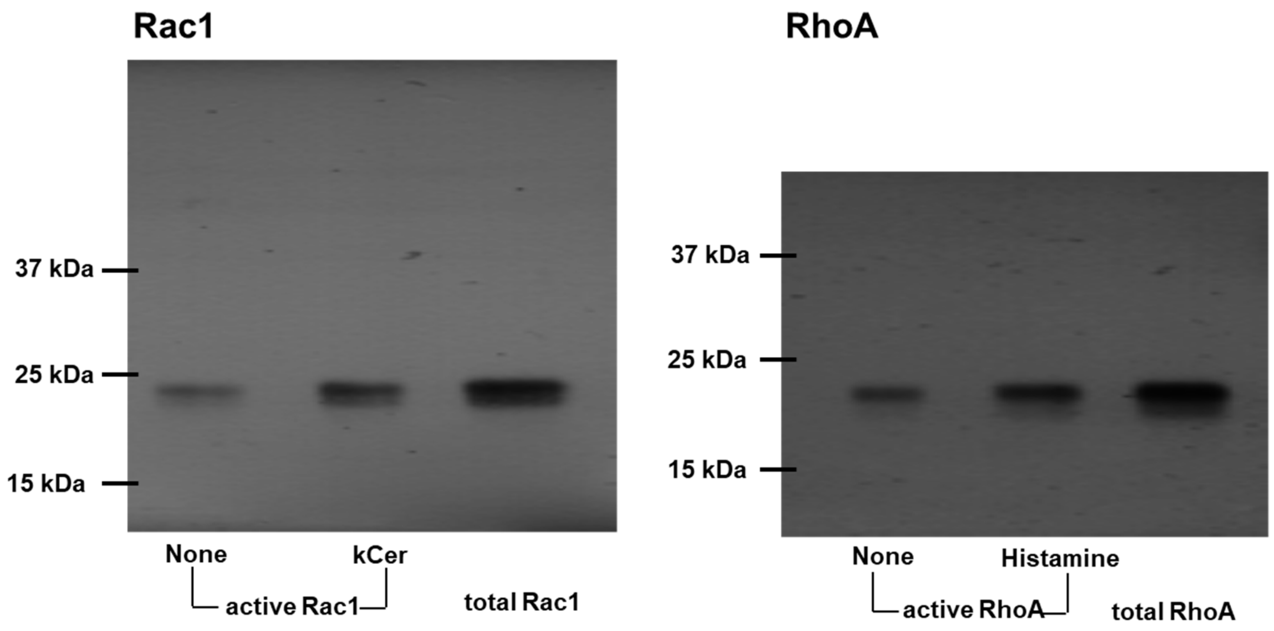

Figure S7.

Supplementary Western blot membrane images of the Rac1 and RhoA activation assay. Cells were treated with 10 μM kCer or 10 μM histamine in L-Ca medium, and cell lysates were then prepared using RIPA Buffer. Active fractions of Rac1 and RhoA were obtained using affinity beads from the G protein assay kit. Total Rac1 and total RhoA are shown in the third lane of each blot.

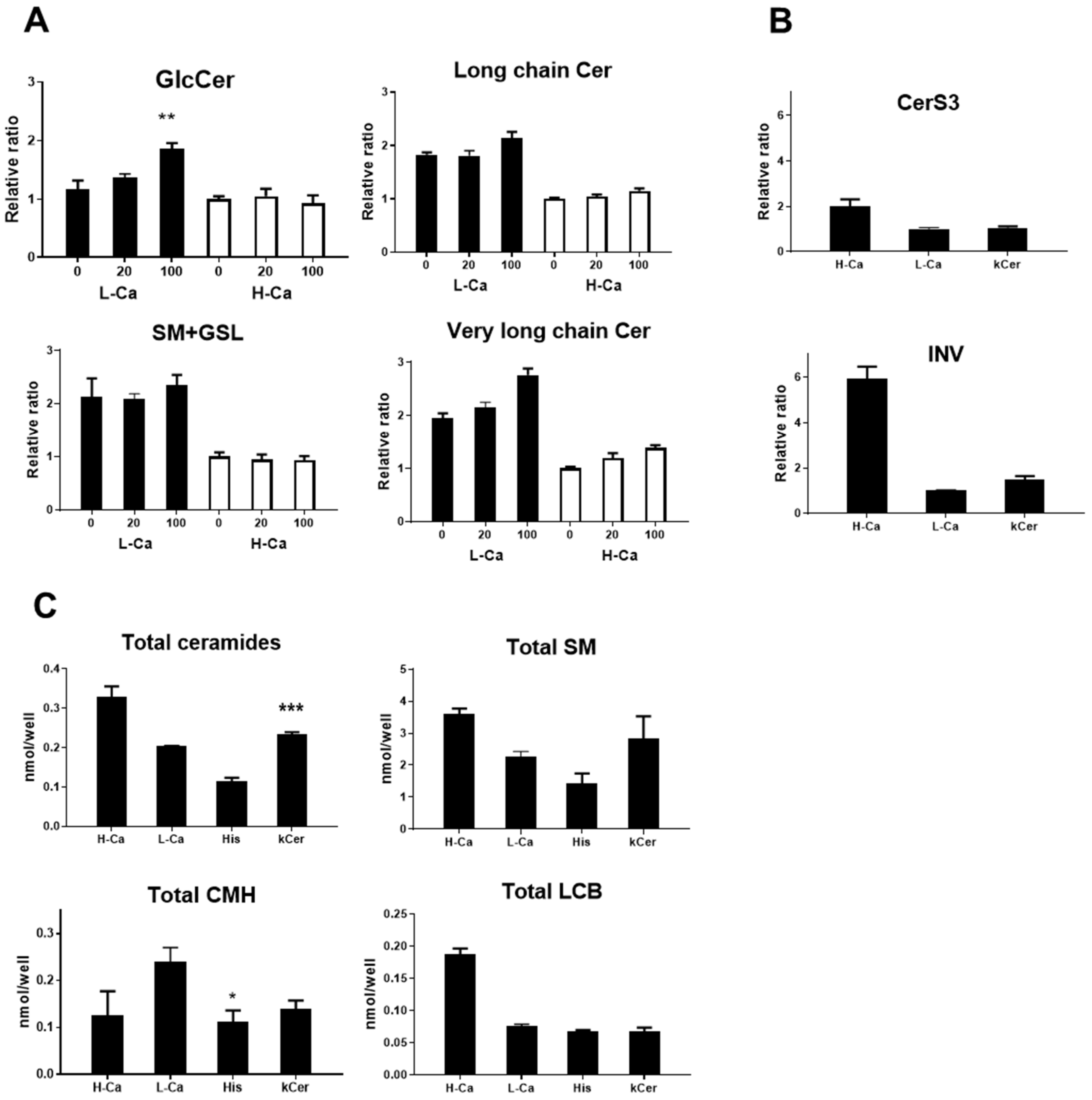

Figure S8.

Regulation of sphingolipid metabolism by cell differentiation. (**A**) HaCaT cells cultured under short-term L-Ca conditions were used for metabolic labeling with [^{3}H]DHS in the presence of kCer (0, 20, and 100 µM) in L-Ca medium or H-Ca medium for 24 h. Lipids were extracted, subjected to thin layer chromatogarphy (TLC), and the desnsities of the spots developed TLC plates were determined by autoradiographic analysis. Values are calculated as percentage of 0 µM kCer, respectively. **p<0.001 vs. no treatment (None), respectively, one-way ANOVA followed by Tukey's multiple comparison post-test and Dunnett's test.

(**B**) HaCaT cells under long-term culture conditions were treated with H-Ca medium, L-Ca medium, or kCer in L-Ca medium for 24 h. Total RNA was then prepared for qPCR analysis. CerS3, ceramide synthase 3; INV, involucrin. Data were expressed as relative ratio of L-Ca samples vaules.

(**C**) HaCaT cells under long-term culture conditions were treated with H-Ca medium, L-Ca medium, 10 μM histamine in L-Ca medium, or 10 μM kCer in L-Ca medium for 24 h. Lipids were then prepared for Bligh-Dyer extraction. Lipid samples were analyzed by LC-MS/MS as described in the Methods section and expressed as nmol lipids per well of a six-well culture plate.
